# Health of Great Cities

**Published:** 1875-01

**Authors:** 


HEALTH OF GREAT CITIES.—Some scientific writers have lately predicted that the great cities of the world would shortly become so large as to become uninhabitable. London is cited as an example. This city now has an estimated population of 3,400,700, and the estimate is made that 600,000,000 cubic feet of carbonic acid gas are respired there every twenty-four hours by human beings alone, and that 14,000 tons of coal are daily consumed there, a great portion of which is cast into the atmosphere in the partially volatilized form of smoke. It is assumed that this impaired air will by and by fail to sustain life. But fortunately for the dwellers in London, and unfortunately for the theories of the croakers, there are many miles of fresh air above the city constantly replacing the vitiated atmosphere of the streets. London may be extended indefinitely, with, in all probability, no perceptible change in the life-supporting power of her atmosphere. The registration of births and deaths in England is so thoroughly made that the estimates of population based thereon very nearly agree with the actual count from the census returns, and such registration has as yet given no signs of any increased ratio of deaths following the growth of London. In this connection the fact may be noted that London contains as much population as the seventeen next principal cities of Great Britain and Ireland, and that, according to the latest estimates, the population of London is increasing in a larger ratio than that of other cities. Nor need New-Yorkers at present flee the city to secure better sanitary influences in the suburbs. There is air enough for all if we will but welcome and not exclude it. The following is the death rate of the chief cities of the civilized world for the year 1873, showing the number of deaths to each thousand inhabitants, according to the returns from official journals:—St. Louis, 19 died out of every 1,000; Philadelphia, 20; London, 22; Cincinnati, 23; Paris, 23; Bombay, 24; Brussels, 25; Calcutta, 25; Brooklyn, 25; Baltimore, 25; New York, 28; Boston, 29; Madras, 36; New Orleans, 37. Families who have a whole house to themselves are healthier and live longer than those who are more crowded: but as half the population of New York lives in tenement houses, that is, from two to twenty families under one roof—the proportion being six persons to one residence in Philadelphia, and fifteen in New York—the latter city does not show as favorable a death-rate as Philadelphia. Philadelphia, in 1870, had dwellings, 112,336; New York, 64,044; Brooklyn, 33,868; St. Louis, 38,384; Baltimore, 27,706; Cincinnati, 13,284. The general inference is that the more roomy a house is, that is, the larger the rooms, especially for sleeping purposes, the more healthy it is. And yet there is a kind of indefinite impression that any place, however small, or cluttered up, is good enough to sleep in, while the fact is that a single sleeper ought to have a room twelve feet square.

---

GENTLEMAN.—Every one who bears the name of a Gentleman is accountable for it to his family.

